# Supplementary material for: Identification of photoperiod-induced specific miRNAs in the adrenal glands of Sunite sheep (Ovis aries)
Source: Front Vet Sci. 2022 Jul 22;9:888207. doi: 10.3389/fvets.2022.888207 (PMC9354845; doi:10.3389/fvets.2022.888207)
Supplement: Supplementary file 1 [file Data_Sheet_1.ZIP › Supplementary file/miRDeep2 file.zip!/miRDeep2 file/Know_Structure/SPLP42A3/oar-mir-3958.pdf]

miRBase precursor : oar-mir-3958
Total read count : 14518
oar-miR-3958-5p read count : 22
oar-miR-3958-3p read count : 14495
remaining reads : 1

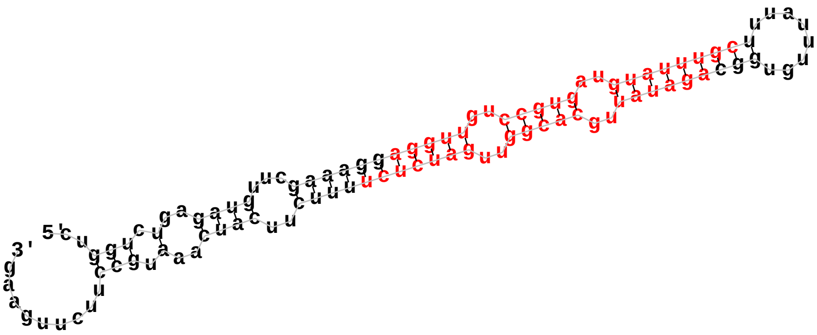

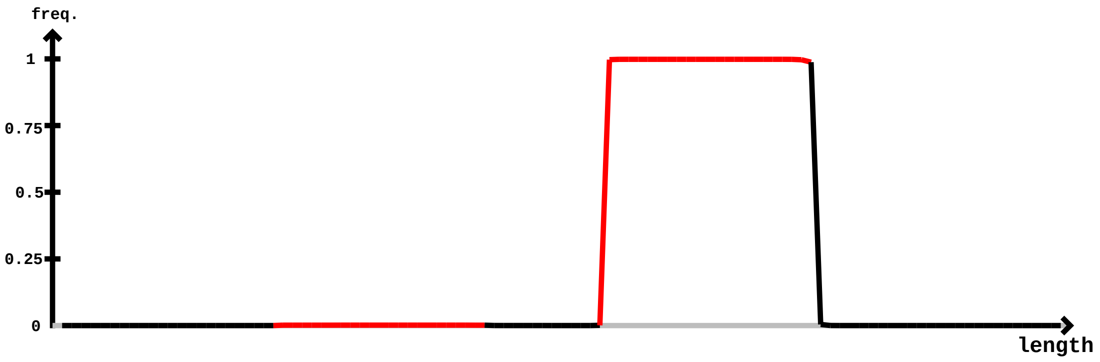


```
                                                                oar-miR-3958-3p
                     oar-miR-3958-5p
5'- cuggucugagauguucgaaaggagguuguccgugauguauuugcuuuauuuguggcagauauugcacgguugaucucuuuucuucaucaaaugccuucuugaag   -3'    exp
    ..(((.(..((((...(((((((((((..(((((..(((((((((........)))))))))..)))))..)))))))))))..))))..).))).........   reads     mm        sample
    ......................agguuguccgugauguauuu..............................................................   2         0         seq
    ......................agguuguccgugauguauuugc............................................................   18        0         seq
    ......................agguuguccgugauguauuugcu...........................................................   2         0         seq
    ............................uccgugauguauuugcuuuauu......................................................   1         0         seq
    .......................................................cagauauugcacgguugaucucu..........................   13        0         seq
    ........................................................agauauugcacgguugauc.............................   3         0         seq
    ........................................................agauauugcacgguugaucu............................   22        0         seq
    ........................................................agauauugcacgguugaucuc...........................   131       0         seq
    ........................................................agauauugcacgguugaucucu..........................   14245     0         seq
    ........................................................agauauugcacgguugaucucuu.........................   59        0         seq
    ........................................................agauauugcacgguugaucucuuu........................   3         0         seq
    .........................................................gauauugcacgguugaucuc...........................   1         0         seq
    .........................................................gauauugcacgguugaucucu..........................   17        0         seq
    ..........................................................auauugcacgguugaucucu..........................   1         0         seq
```
